# *In vivo* Biocompatibility and Bioactivity of Calcium Silicate-Based Bioceramics in Endodontics

**DOI:** 10.3389/fbioe.2020.580954

**Published:** 2020-10-29

**Authors:** Wencheng Song, Wei Sun, Lili Chen, Zhenglin Yuan

**Affiliations:** ^1^Department of Stomatology, Union Hospital, Tongji Medical College, Huazhong University of Science and Technology, Wuhan, China; ^2^Hubei Province Key Laboratory of Oral and Maxillofacial Development and Regeneration, Wuhan, China

**Keywords:** *in vivo*, calcium silicate, bioceramics, biocompatibility, bioactivity, endodontics

## Abstract

Endodontic therapy aims to preserve or repair the activity and function of pulp and periapical tissues. Due to their excellent biological features, a substantial number of calcium silicate-based bioceramics have been introduced into endodontics and simultaneously increased the success rate of endodontic treatment. The present manuscript describes the *in vivo* biocompatibility and bioactivity of four types of calcium silicate-based bioceramics in endodontics.

## Introduction

Mineral trioxide aggregate (MTA) was first introduced into endodontics as calcium silicate-based bioceramic for root-end filling and displayed great biocompatibility and bioactivity to surrounding cells/tissues. Subsequently it was recommended to be a potential material for pulp capping, apexogenesis, and other endodontic applications (Tawil et al., 2015). After that, a variety of calcium silicate-based bioceramics have been developed and applied into endodontics, such as Bioaggregate, Biodentine, and iRoot, which are the most commonly used calcium silicate-based bioceramics in endodontics. Besides, there are also some other calcium silicate-based bioceramics which are rarely applied in endodontics, such as Endo CPM Sealer (EGO SRL, Buenos Aires, Argentina), BioRoot RCS (Septodont, France), and TechBiosealer (Profident, Kielce, Poland). Therefore, the present review mainly describe the biocompatibility and bioactivity of these four types of calcium silicate-based bioceramics: MTA, Bioaggregate, Biodentine, and iRoot. Compared to MTA, these novel calcium silicate-based bioceramics possess comparable biological characteristics in terms of low cytotoxicity, mild inflammation response, and superior capacity to promote cell viability and tissue repair (de Oliveira et al., 2018). In addition, calcium silicate-based bioceramics could function as human tissues, as well as encourage a regenerative response in natural tissues such as osteoinduction, which is similar to that of hydroxyapatite (Raghavendra et al., 2017). Therefore, the biocompatibility and bioactivity of these calcium silicate-based bioceramics was closely related to the outcome and efficiency in endodontic applications, such as pulp capping, root-end filling, perforation repair, and pulp regeneration. Various *in vitro* studies which described the physicochemical and biological properties of these bioceramics, have been summarized in a wide range of previous reviews (Al-Haddad and Che, 2016; Raghavendra et al., 2017; Primus et al., 2019). However, there have been few reviews about the *in vivo* studies of calcium silicate-based bioceramics in endodontics. So, this review will focus on the *in vivo* performance of these bioceramics on different animal models and provide a reference and guideline for future research.

Animal models could be performed to mimic the human body reaction to these bioceramics in endodontics. Therefore, various animal models were created to assess the biocompatibility and bioactivity of calcium silicate-based bioceramics on tissues or organs. Low cost, ease of handling, and homogeneity of the genetic background are the advantages of utilizing rodent animals widely. However, larger animals have a higher degree of similarity to the human body and the size of their teeth is closer to that of human teeth. So larger animals are considered to be more suitable to ensure the accuracy and significance of the results, especially when operating in the root canal space (Nakashima et al., 2019). In addition, the inherent heterogeneity of animal subjects could lead to inconsistent results (Robinson et al., 2019). Therefore, selecting appropriate animal models is essential to evaluate the performance of advancing regenerative biomaterials in mechanistic or pre-clinical trials. Although these animal models are widely used, it is worth noting for the researcher that no model could replicate the complex human response induced by this biomaterial and animal models should be just used to provide a significant number of experiment data and direction toward the human studies (Zhan et al., 2016).

The aim of this review is to summarize the studies about the *in vivo* biocompatibility and bioactivity of four common calcium silicate-based bioceramics in endodontics via different animal models, including subcutaneous implantation, dental pulp capping, root perforation repair, root-end filling, regenerative endodontic procedures, and apexification. Moreover, several common animals, including monkey, sheep, dog, rat, ferret, and mouse, have been selected to create the animal models in endodontics, which will also be summarized and discussed in this review. All the related information including the text and references have been summarized in Table 1. The present review will provide guidance for the application of these calcium silicate-based materials in endodontics based on the summary information about their *in vivo* biocompatibility and bioactivity. In addition, the composition, production, major advantages and disadvantages, as well as their application fields in endodontics of these four calcium silicate-based bioceramics are provided in Table 2.

**TABLE 1:** Biocompatibility and bioactivity of calcium silica te-based bioceramics in endodontics: *in vivo* studies.

Animal model	Calcium silicate- based bioceramics	Species	Biocompatibility/bioactivity	References
Subcutaneous implantation	MTA	Rat	• Severe/moderate inflammation on day 7 and decreased over time	Shahi et al., 2010; Cintra et al., 2013; Bueno et al., 2019
			• Thick and loose fibrous capsule formation on day 7 and was replaced by thin and dense fibrous capsule formation finally	Cintra et al., 2013; Taha et al., 2016; Bueno et al., 2019
			• The thickness of fibrous capsules increased over time	Khalil and Abunasef, 2015
			• Dystrophic calcification and birefringent structure	Viola et al., 2012; Cintra et al., 2013; Hinata et al., 2017; Bueno et al., 2019
			• Enhanced M2 macrophage polarization	Ito et al., 2014
		Mouse	• Induced the acute inflammation and biomineralization simultaneously	Reyes-Carmona et al., 2010, 2011
	Bioaggregate	Rat	• Less inflammatory response and produce less calcification compared to MTA	Batur et al., 2013
			• More inflammatory response compared to MTA	Saghiri et al., 2013b
			• Similar biocompatibility to MTA	Bosio et al., 2014
			• Adverse effect on liver function and kidney function	Khalil and Eid, 2013; Simsek et al., 2016
	Biodentine	Rat	• Intense inflammation on day 7 and decreased over time	Mori et al., 2014; Pinheiro et al., 2018
			• Fibrous capsules formation	Da et al., 2016, 2019
			• Similar biocompatibility to MTA or Bioaggregate	Simsek et al., 2015
			• Induce the biomineralization	Martins et al., 2016; Cosme-Silva et al., 2019
	iRoot BP Plus	Rat	• Initiate thick inflammatory capsule containing focal calcification and marked fibrosis	Abou et al., 2019
	iRoot SP	Rat	• Induce inflammatory cell infiltration especially macrophages and multi-nucleated giant cells	Bosio et al., 2014; Zhang and Peng, 2015
Dental pulp capping	MTA	Mouse	• The first 2 days was the inflammatory phase and dentin bridge with strong DSPP expression in odontoblast-like cells at 5 weeks	Nirschl and Avery, 1983
		Rat	• Few inflammatory cell infiltration and mild hard deposition in the first week and dentin bridge was induced at 4 weeks	Kramer et al., 2014; Park et al., 2014; Chang et al., 2016; Han et al., 2017; Long et al., 2017
			• Increased expression of odontogenic-related genes, such as DSPP, DMP1, and ON	Park et al., 2014
			• Inhibit the expression of proinflammatory cytokines IL-1α and IL-1β	Kramer et al., 2014
			• KLF5 was expressed in odontoblast-like cells and dental pulp cells	Han et al., 2017
			• Glut2 and Glut4 were expressed in differentiated odontoblast-like cells	Tohma et al., 2020
		Dog	• Display better performance in terms of pulp viability, pulp inflammation, and calcified bridge formation	Tabarsi et al., 2010
			• Induce the produce of odontoblast-like cells	Tabarsi et al., 2010
			• Induce the formation of reparative dentin with irregular features	Tziafas et al., 2000
	Bioaggregate	Rat	• Dentin bridge was thinner and the density of reparative dentin in MTA was lower than that in MTA or Biodentine	Kim et al., 2016
	Biodentine	Mouse	• Promote the differentiation of bone marrow-derived cells into odontoblast-like cells	Frozoni et al., 2020
		Rat	• Induce the formation of mineralized tissue aggressively compared to MTA	Paula et al., 2020
			• Induce the activation of Wnt/β-catenin for dentin bridge formation	Yaemkleebbua et al., 2019
		Dog	• Less inflammatory response and more dentin bridge formation compared to MTA	Zaen et al., 2020
	iRoot BP Plus	Rat	• Exhibit mild inflammation and induce the dentin bridge formation	Liu et al., 2015; Okamoto et al., 2018
			• Induce stronger expression of odontogenic and focal adhesion molecules beneath the dentin bridge	Zhu et al., 2014a; Zhang et al., 2015
		Dog	• Complete calcified bridge formation without pulp inflammation	Shi et al., 2016
Root perforation repair	MTA	Rat	• Increased number of polymorphonuclear cells and mononuclear cells, abundant collagen deposition and granulation tissue	Silva et al., 2009
			• Decrease the inflammatory response and the bone resorption	de Sousa et al., 2019
		Mice	• Increase the expression of pro-inflammatory cytokines	Lara et al., 2015
		Dog	• Induce mild inflammation and the formation of hard tissue bridge and inhibit the epithelial infiltration	Holland et al., 2001; Yildirim et al., 2005; Samiee et al., 2010
			• Delayed application of MTA in perforation led to the contamination of the perforation site	Ford et al., 1995; Tawfik et al., 2016
	Biodentine	Rat	• Decrease the inflammatory response and the bone resorption	de Sousa et al., 2019
		Dog	• Induce the formation of new mineralized tissue without bone resorption and inflammatory cell infiltration	Silva et al., 2017; Cardoso et al., 2018
Root-end filling	MTA	Dog	• Less inflammatory infiltration and more fibrous capsule underneath MTA, the deposition of new cementum	Torabinejad et al., 1995; Baek et al., 2005; Bernabe et al., 2005; Otani et al., 2011; Walivaara et al., 2012
			• Enhance the regeneration of cementum, bone and periodontal ligament with less inflammatory infiltration	Regan et al., 2002; Tanomaru-Filho et al., 2006; Tawil et al., 2009; Baek et al., 2010; Kohout et al., 2015; Walsh et al., 2018
		Monkey	• No inflammatory response and induce the cementum formation	Torabinejad et al., 1997
	Biodentine	Dog	• Display stronger sealing ability than MTA, promote periradicular bone healing	Tang et al., 2019
Regenerative endodontic procedures	MTA	Dog	• Induce the formation of bone-like tissue, cementum-like tissue, and periodontal ligament-like tissue	Wang et al., 2010; Zhang et al., 2014; Rodriguez-Benitez et al., 2015; Saoud et al., 2015; Moradi et al., 2016; Stambolsky et al., 2016; Ghoddusi et al., 2017; Palma et al., 2017
		Sheep	• Induce the formation of bone-like tissue, cementum-like tissue, and periodontal ligament-like tissue	Altaii et al., 2017
		Ferret	• Induce the formation of bone-like tissue, cementum-like tissue, and periodontal ligament-like tissue	Torabinejad et al., 2014, 2015, 2018
Apexification	MTA	Dog	• Induce aical closure, hard tissue formation, and less inflammatory infiltration	Shabahang et al., 1999
			• Induce the resolution of periapical lesion and apical closure	Ham et al., 2005
		Monkey	• Induce aical closure, hard tissue formation, and less inflammatory infiltration	

**TABLE 2:** Classification of calcium silicate-based bioceramics in endodontics.

Calcium silicate-based bioceramics	Producers	Compositions	Advantages	Disadvantages	Major application fields
MTA	Dentsply Endodontics, Tulsa, OK, United States	Tricalcium silicate, dicalcium silicate, tricalcium aluminate, tetracalciumaluminoferrite, calcium sulfate, and bismuth oxide	• Excellent sealing ability and stability • Better adaptation to the dentinal walls	• Long setting time, difficult handling characteristic and teeth discoloration. • Weaker bonding or retentive strength.	• Apexification. • Root perforation and resorption. • Pulp capping. • Root end filling and sealing. • Regenerative endodontic procedures.
Bioaggregate	Innovative Bioceramix Inc., Vancouver, Canada	Tricalcium silicate, tantalum oxide, calcium phosphate, silicon dioxide	• Better color stability than MTA. • More stable bond strength than MTA. • Higher fracture resistance and acid resistance than MTA. • Lower washout than Biodentine. • High specific surface area.	• Inferior mechanical properties and bond strength than MTA. • Higher fluid uptake and longer setting time than Biodentine.	• Apexification. • Root perforation and resorption. • Pulp capping. • Root end filling and sealing. • Regenerative endodontic procedures.
Biodentine	Septodont, Saint Maur des Fosses, France	Tricalcium silicate, zirconium oxide, calcium carbonate	• Superior mechanical properties than Bioaggregate. • More stable dimension than MTA. • Higher bond strength and least discoloration possibility. • Excellent ability to resist dislodgement and root fracture. • Good sealing ability.	• Unfavorable radiopacity and very high washout tendency. • Weaker antibacterial and anticariogenic effects than glass ionomer cement.	• Root end filling and perforation repair. • Pulpotomy procedures. • Pulp capping. • Regenerative endodontics.
iRoot BP/BP plus	Innovative Bioceramix Inc., Vancouver, Canada	Calcium silicate, calcium phosphate, and aluminum	• Easy manipulation and faster setting time than MTA.	• Inferior sealing capacity than MTA.	• Root canal filling and repair.
iRoot FS	Innovative Bioceramix Inc., Vancouver, Canada	Calcium silicates, zirconium oxide, tantalum pentoxide, calcium phosphate monobasic, anhydrous calcium sulfate	• Shorter setting time and hydrating process than MTA. • Equal compressive strength and microhardness.		• Root canal repair and filling.
iRoot SP	Innovative Bioceramix Inc., Vancouver, Canada	Calcium silicate, calcium phosphate, zirconium oxide, tantalum oxide.	• Advantageous penetration area than MTA.	• Weaker bond strength than MTA.	• Root canal filling and sealing.

## Classification of Calcium Silicate-Based Bioceramics in Endodontics

### Mineral Trioxide Aggregate (MTA)

Mineral trioxide aggregate (MTA) is mainly composed of calcium and silicate elements and was first applied in endodontic therapy as calcium silicate-based bioceramics by Dr. Torabinejad in 1993 (Lee et al., 1993). Considering that tooth discoloration was caused by gray MTA (GMTA), white MTA (WMTA) was introduced to work out this problem by reducing the concentration of FeO in 2002 (Dammaschke et al., 2005; Emine and Tuba, 2011). Nevertheless, recently it was reported that the interaction of bismuth oxide with collagen present in teeth along with sodium hypochlorite and chlorhexidine used during root canal therapy are more likely to be the main reasons for discoloration (Camilleri, 2014; Marciano et al., 2014; Niu et al., 2015). To prevent the discoloration caused by bismuth oxide, other substances such as calcium tungstate or zirconium oxide were used to replace bismuth oxide (Duarte et al., 2018; Aly et al., 2019). Compared with traditional materials, the bonding or retentive strength of MTA was remarkably weaker. However, flexural strength of MTA dramatically increases after 24 h. Besides, the sealing ability and stability depend on the thickness of MTA. Therefore, at least 3 mm of MTA should be provided when used as an apical restoration or repair of root perforation (Roberts et al., 2008; Surya Raghavendra et al., 2017). The excellent sealing capacity of MTA may be associated with its initial mechanical seal and the subsequent formation of hydroxyapatite crystals which is induced by the dissolved production of MTA and able to react with dentine to get a chemical adhesion (Sarkar et al., 2005). Exposure of MTA to a range of acidic environments might have negative effects on the sealing ability (Saghiri et al., 2008). Besides, MTA exhibits a better adaptation to the dentinal walls than amalgam, due to the fine hydrophilic particles contained in MTA that absorbs water and contributes to the expansion of materials during hydration (Shipper et al., 2004; Badr, 2010). Although MTA is one of the most popular calcium silicate-based bioceramics in endodontics with lots of advantages, it also has some drawbacks including unsatisfactory setting time, difficult handling characteristics, and the risk of teeth discoloration (Camilleri, 2015; Surya Raghavendra et al., 2017).

### Bioaggregate

Bioaggregate (Innovative Bioceramix Inc., Vancouver, Canada), which has similar chemical constituents to MTA but with some differences, is almost free of hazardous substances such as aluminum and bismuth oxide which is replaced by tantalum oxide as a radiopacifier. Different to bismuth oxide, tantalum oxide is inert and tantalum can not be released into the solution (Park et al., 2010; Camilleri et al., 2015). As a result, Bioaggregate displayed better color stability than MTA when immersed in sodium hypochlorite or chlorhexidine gluconate (Keskin et al., 2015). Besides, silicon dioxide and calcium phosphate contained in Bioaggregate were able to promote the formation of calcium hydroxide, and calcium ion released early during the hydration reaction could remain in a very alkaline pH environment for remineralization during a 28-day period (Camilleri et al., 2015). Differences in their hydration kinetics may contribute to the various bond strength and mechanical performances of calcium silicate-based bioceramics. Compared with MTA, hydration of Bioaggregate produces more calcium silicate hydrate (C-S-H) and the amorphous nature of hydroxyapatite (HA) which are both poor-crystalline nanometer-level structures, but rarely the formation of portlandite. So the mechanical properties and bond strength of Bioaggregate are inferior to MTA (Hashem and Wanees, 2012; Schembri-Wismayer and Camilleri, 2017). Nevertheless, Bioaggregate shows more stable bond strength than MTA when it acts as coronal plugs (Amin and Gawdat, 2018). Meanwhile, Bioaggregate reveals higher fracture resistance (Tuna et al., 2011; Guven et al., 2016) and acid resistance than MTA (Hashem and Wanees, 2012), while showcasing lower washout, higher fluid uptake, and longer setting time than Biodentine (Grech et al., 2013b). In addition, Bioaggregate displays a comparable sealing ability with MTA (Leal et al., 2011). The thicknesses of apical plugs and different irrigation agents are reported to affect the apical leakage. Twelve millimeters of Bioaggregate exhibits the best resistance to leakage while Ethylenediaminetetraacetic acid (EDTA) and a detergent increase the apical leakage of Bioaggregate. Besides, considering that chlorhexidine does not seem to influence the sealing performance, it becomes more popular in endodontic applications (El Sayed and Saeed, 2012; Bayram et al., 2015; Memis et al., 2015). Furthermore, the bioceramic porosity could not only affect its resistance capacity to leakage (Saghiri et al., 2008), but also affect the adhesion of surrounding cells (Chen et al., 2009). Bioaggregate is characterized by a high specific surface area which contains homogenous, round, and small particles, and therefore is regarded as a calcium silicate-based bioceramic with superior physicochemical properties (Camilleri et al., 2015; Chang, 2018).

### Biodentine

Biodentine (Septodont, Saint-Maur-des-Fossés, France) serves as a representative of the tricalcium silicate-based bioceramics and some properties of its ingredients formulated by the MTA-based bioceramics have been improved (Grech et al., 2013a). Biodentine does not contain calcium aluminate and calcium sulfate, but these two ingredients exist in MTA which cause decreased mechanical strength and a longer setting time (Caron et al., 2014). Meanwhile, compared to Bioaggregate, Biodentine also exhibited superior mechanical properties including compressive strength and microhardness (Grech et al., 2013b). In addition, Biodentine showed a more stable dimension than MTA and a 0.58% loss in volume (Petta et al., 2020). Due to advantages including stronger microstructure, higher bond strength, and lower discoloration possibility, Biodentine has become an excellent candidate in endodontics compared to Bioaggregate and MTA (Bortoluzzi et al., 2009; Grech et al., 2013b; Alsubait et al., 2014; Malkondu et al., 2014; Camilleri, 2015; Yoldas et al., 2016; Majeed and AlShwaimi, 2017). However, the microhardness values of both Biodentine and MTA was decreased under acidic pH conditions, which led to the formation of more porous but less crystalline microstructures (Bolhari et al., 2014; Deepthi et al., 2018). Biodentine with available thickness displayed an excellent ability to resist dislodgement (Zhu et al., 2014b; Ulusoy et al., 2016) and root fracture (Ulusoy and Paltun, 2017) compared to Bioaggregate and MTA, whereas excessive thickness had adverse effects on fracture resistance (Eram et al., 2020). Moreover, Biodentine with a thickness of 4 millimeters was able to provide an optimal apical seal and marginal adaptation (Brito-Junior et al., 2014). Besides, the dentin bond strength of repair materials was important to maintain the integrity of the sealer in endodontics. Like other calcium silicate-based bioceramics, the bond strength of Biodentine could be affected by substances used in the procedure of root canal preparation, such as irrigants, chelating agents, and acids (Ballal et al., 2018). For instance, the bond strength of Biodentine was suitably reinforced when the root canal was irrigated with a mixture of NaOCl and 1-hydroxyethane 1,1-Diphosphonic (HEDP) than that with NaOCl alone (Paulson et al., 2018). NaOCl and saline solutions had a similar effect on the bond strength of Biodentine (Guneser et al., 2013), and increased its sealing capacity while EDTA significantly enhanced the microleakage of Biodentine (Al-Azzawi and Al-Zubaidi, 2014). Given the good sealing ability and excellent biological properties in Biodentine, it could be applied in retrograde-filling in endodontics as root-end filling materials (Solanki et al., 2018; Nabeel et al., 2019; Tang et al., 2019). However, the sealing ability of calcium silicate-based bioceramics could be influenced by wettability, blood conditions, and temperature ranges occurring in the oral cavity instead of the effect of surface roughness and vertical dimensional changes of the materials (Saghiri et al., 2013a; Aksel et al., 2018; Singla et al., 2018). Beyond that, the acidic periapical environment could affect the sealing ability of Biodentine in endodontics via promoting its solubility (Pushpa et al., 2018). Despite Biodentine’s desirable properties, it revealed unfavorable radiopacity and a very high washout tendency (Grech et al., 2013b; Caron et al., 2014). The antibacterial and anticariogenic effects of Biodentine were weaker than glass ionomer cement due to the lack of fluoride ion which was able to inhibit plaque bacteria formation and drive remineralization. Therefore, bioactive glass could be added into Biodentine to promote the apatite formation (Forss et al., 1991; Simila et al., 2018).

### iRoot BP/FS/SP

iRoot BP/BP Plus (Innovative Bioceramix Inc., Vancouver, Canada) are novel calcium silicate-based bioceramics in endodontics developed for permanent root canal repair and filling applications, which have similar composition and mainly consist of calcium silicate, calcium phosphate, and aluminum. The manufacturer have claimed that their physical and mechanical characteristics were equal to those of MTA. Nevertheless, Onay et al. (2014) reported that iRoot BP possessed inferior sealing capacity compared to MTA via the fluid filtration method and scanning electronic microscopy evaluation. However, easy manipulation and faster setting time are the highlights of iRoot BP/BP Plus when compared to MTA. The setting time of iRoot BP/BP Plus is 2 h while the setting time is 4 h for MTA. iRoot Fast Set (iRoot FS, Innovative Bioceramix Inc., Vancouver, Canada) is another nanoparticle bioceramic which has similar ingredients with iRoot BP/BP Plus (Yang, 2008; De-Deus et al., 2012). In comparison with MTA, the setting time and hydrating process of iRoot FS was shorter (Guo et al., 2016), but the compressive strength and microhardness of iRoot FS and MTA were relatively equal (Guo et al., 2016). It has been proven that adequate bond strength could be obtained in iRoot FS at the initial setting time (20 min), which was comparable with iRoot BP and MTA at either the initial setting point or 7 days after setting (Dong et al., 2018). Besides, iRoot FS had comparable apical sealing potential compared to MTA, which suggested that iRoot FS could be used in root-end filling (Shi et al., 2015). iRoot SP (Innovative Bioceramix Inc., Vancouver, Canada) is a premixed calcium silicate-based bioceramic paste for root canal sealing application, which could penetrate into the dentinal tubule and then create excellent mechanical interlocking between iRoot SP and dentin (Haragushiku et al., 2010). iRoot SP exhibits an advantageous penetration area (Akcay et al., 2016) but a weaker bond strength (Oliveira et al., 2016) compared to AH Plus (Dentsply De Trey Gmbh, Konstanz, Germany) or MTA. In addition, iRoot SP has an equivalent apical sealing ability with the AH Plus sealer (Zhang et al., 2009). The moisture of a root canal is an element related to its setting and sealing effect. So the setting process of iRoot SP may be prolonged if the application site in the teeth is dry and iRoot SP has the best sealing ability when the root canal area is slightly moist (Chen et al., 2018).

## Subcutaneous Implantation

Subcutaneous implantation is a regular method to assess the biocompatibility of bioceramics *in vivo* when applied in endodontics. Generally, surgical cavities are made in the animal’s back and then polyethylene tubes filled with the bioceramics are implanted into the surgical cavities. After that the biopsies are obtained for hematoxylin-eosin staining and then the observation of the cellular and inflammatory events are used to evaluate the biocompatibility of the bioceramics *in vivo*.

Mineral trioxide aggregate induced dense and severe inflammatory cell infiltration at 7 days after subcutaneous implantation in Sprague-Dawley rats while the inflammation decreased over time, and finally there was no inflammation at 90 days in the presence of MTA (Shahi et al., 2010). According to previous reports, a material that initiated the inflammation but subsided over time could be considered as a biocompatible material (Hauman and Love, 2003). To improve the biocompatibility of MTA, MTA mixed with disodium hydrogen phosphate (Na_2_HPO_4_) led to a mild inflammation reaction after 7 and 15 days compared to MTA alone (Lotfi et al., 2009). The mild inflammation in the presence of Na_2_HPO_4_ might be due to the formation of hydroxyapatite caused by the interaction of MTA and Na_2_HPO_4_ (Sarkar et al., 2005). In addition, MTA could not only induce the inflammatory response but also the fibrous capsule formation. It was found that MTA induced a moderate inflammation infiltrate and thick fibrous capsule formation 7 days after the subcutaneous implantation of MTA in Wistar rats, but the inflammation response subsided over time and finally was replaced by a thin fibrous connective tissue capsule after 30 and 60 days (Cintra et al., 2013; Bueno et al., 2019). Besides, MTA also produced dystrophic calcification and birefringent structures after 7 and 30 days, which suggested that MTA was biocompatible and also able to promote the biomineralization, which was consistent with the previous study that the subcutaneous implantation of MTA in Wistar rats produced apatite-like surface precipitates containing Ca and P, as well as a thick Ca- and P-rich layers at the material-tissue interface after 7 days (Hinata et al., 2017). The biomineralization induced by MTA could be explained by the fact that osteopontin was obviously expressed in the fibroblast cytoplasm of the fibrous capsule and osteopontin was essential to the initial bone matrix formation and calcification (Viola et al., 2012).

In terms of the fibrous capsule formation, it was shown that, after subcutaneous implantation in Wistar rats, MTA induced the initiation of thick capsule formation which contained cellular immature loose fibrous tissue at 1 week, while inducing dense fibrous tissue at 6 weeks (Taha et al., 2016). On the contrary, Khalil WA et al. found that the thickness of fibrous capsules induced by MTA increased over time, which might be due to the proliferation of fibroblasts induced by mast cells (Khalil and Abunasef, 2015). Some studies considered that the amount of fibrous capsules was inversely associated with a material’s biocompatibility and that the fibrosis was caused by inflammation (Mussel et al., 2003; Shahi et al., 2006). On the contrary, other studies proposed that the formation of fibrous capsules indicated the tissue tolerance to the implanted material (Yaltirik et al., 2004; Parirokh and Torabinejad, 2010). The contradiction might be attributed to the method chosen to assess the histological parameter. For example, Vosoughhosseini et al. (2012) compared the differences of two histopathologic methods of Cox and the Federation Dentaire International (FDI) to evaluate the subcutaneous reaction of MTA in Wistar rats. It was proven that FDI method is more reliable than the Cox method to evaluate the inflammation.

To further clarify the underlying mechanism on how the early inflammation response induced by MTA affects the biomineralization and wound healing, Reyes-Carmona et al. (2010, 2011) performed subcutaneous implantation in mice and the results showed that the expression of pro-inflammatory cytokines was upregulated during the first 3 days. In addition, the apatite-like clusters on collagen fibrils were also observed at 12 h after implantation by scanning electron microscopic examination. It suggested that MTA induced the acute inflammatory response and the biomineralization simultaneously, and then a series of signaling pathways and cellular events were activated to promote the production of an apatite-like layer and the integration of MTA into surrounding tissue. Besides, Ito et al. (2014) found that, when implanted in subcutaneous tissue in Wistar rats, MTA could potentially enhance M2 macrophage polarization, which suggested that MTA provided a wound healing environment to achieve good biocompatibility and biomineralization via the enhancement of M2 macrophage polarization.

Due to the fact that lots of research has been carried out to investigate the biocompatibility of MTA in subcutaneous tissue, the biocompatibility of other novel bioceramics was usually assessed with a comparison with MTA. Batur et al. (2013) found that Bioaggregate showed less of an inflammatory response and foreign body reaction than MTA. However, Bioaggregate had the superior ability to produce dystrophic calcification in comparison with MTA. Conversely, Saghiri et al. (2013b) reported that, compared to MTA, Bioaggregate displayed more of an inflammatory response at 7, 14, 28, and 60 days after implantation, which could be due to the effect of aluminum compounds contained in MTA on the insolubility of MTA cement (Poggio et al., 2007). In contrast, Bosio et al. (2014) found that Bioaggregate and MTA had similar biocompatibility at 7, 15, 30, and 90 days after subcutaneous implantation in rats. However, both Bioaggregate and MTA were considered to be biocompatible because, no matter what difference existed, the commonality between these studies was that the inflammatory response was initiated in the early period after implantation and then subsided over time. Furthermore, to measure the systematic toxic effect of Bioaggregate and MTA, Bioaggregate or MTA was implanted into rat subcutaneous tissue and liver/kidney function was examined with a blood and histopathological examination. The result discovered that both Bioaggregate and MTA had adverse effects on liver function and kidney function after 7 and 30 days, and MTA had a more severe toxic effect than Bioaggregate (Khalil and Eid, 2013), which could be due to the high levels of chromium and magnesium elements in the liver and kidney released from MTA or Bioaggregate after subcutaneous implantation in Wistar rats (Simsek et al., 2016).

In terms of Biodentine, Pinheiro et al. (2018) examined the biocompatibility of MTA and Biodentine when implanted into Wistar rat subcutaneous tissue. The results revealed that MTA and Biodentine initiated an inflammatory infiltrate at 7 days and the inflammation response gradually decreased during the 90-day period. The fact that the inflammation response happened in the early stage may be caused by calcium ions and the high pH values during setting. Compared to MTA, Biodentine induced a stronger inflammatory response at 7 days but the difference between them disappeared at 90 days, as the intense inflammatory response induced by Biodentine at 7 days decreased gradually and finally the inflammatory response was replaced by a fibrous capsule which contained collagen and fibroblasts. In the meanwhile, Mori et al. (2014) also found that Biodentine initiated a stronger inflammatory response compared to MTA at 7 days post-implantation in Wistar rats, but the inflammatory response between them became similar at 30 days. The results were consistent with another study which discovered that the acute inflammation response induced by Biodentine was stronger than MTA, but in the end both of them exhibited similar fibrous capsule formation, which might be associated with the role of IL-6 that mediated the transition from acute inflammatory response to chronic inflammatory response (Da et al., 2016). However, it was proven that the number of fibroblasts and the collagen content contained in the fibrous capsules induced by MTA was significantly higher than that by Biodentine (Da et al., 2019), which suggested that the collagen formation in response to Biodentine was slower than MTA and the difference was related to the distinction of the physiochemical characteristics between Biodentine and MTA. It was also shown that fibroblast growth factor-1 and mast cells participated in fibrous capsule formation via stimulating fibroblast proliferation and collagen production when Biodentine or MTA was implanted into subcutaneous tissue in rats (Da et al., 2019). Simsek et al. (2015) tested the biocompatibility of Biodentine, MTA, and Bioaggregate in Wistar rats and it was proven that the biocompatibility of MTA and Bioaggregate was similar at 7 days while Biodentine was more biocompatible than MTA and Bioaggregate. However, MTA, Bioaggregate, and Biodentine had similar biocompatibility *in vivo* at 45 days. In addition to biocompatibility, Cosme-Silva et al. (2019) found that both MTA and Biodentine had a similar potential to induce the biomineralization at 7 and 30 days after subcutaneous implantation in rats. In the meanwhile, it was found that hypertension could increase the inflammatory infiltrate and decrease the biomineralization induced by MTA or Biodentine (Martins et al., 2016; Cosme-Silva et al., 2019). However, another kind of systemic disease, diabetes mellitus did not influence the biocompatibility and biomineralization of MTA in subcutaneous tissue in Wistar rats (Gomes-Filho et al., 2015; Gomes et al., 2016).

In terms of iRoot BP/SP, Abou et al. (2019) reported that iRoot BP Plus initiated the production of thick inflammatory capsules containing focal calcification and marked fibrosis while MTA induced a tiny fibrin clotted area at 1 week after subcutaneous implantation in Wistar rats. iRoot BP Plus could induce thicker and looser fibrous capsules while MTA induced thin and dense fibrous capsules at 4 weeks (Abou et al., 2019). Zhang and Peng (2015) reported that MTA and iRoot SP had similar biocompatibility on day 7, 30, and 60 days after rat subcutaneous implantation. However, Bosio et al. (2014) discovered that, compared to MTA, iRoot SP displayed a stronger inflammatory response and more inflammatory cells especially macrophages and multi-nucleated giant cells after implantation in Wistar rats.

## Dental Pulp Capping

Dental pulp capping was performed by the application of bioceramics in the deep carious cavity to reverse pulp inflammation, promote pulp regeneration, and preserve the viability of pulp tissue. Whether there was pulp exposure or not, dental pulp capping is classified into direct pulp capping and indirect pulp capping (Kunert and Lukomska-Szymanska, 2020). Since calcium hydroxide (CH) was reported to be successfully used in pulp capping between 1934 and 1941 (Fava and Saunders, 1999), CH was considered as the gold standard in pulp capping. However, CH possessed some disadvantages, such as poor sealing ability, dissolution over time, and weak adherence to dentin (Kunert and Lukomska-Szymanska, 2020). So nowadays CH has been gradually replaced by calcium silicate-based bioceramics especially MTA in the pulp capping procedure. So far there have been plenty of studies to investigate the application of MTA in pulp capping.

When applied in direct pulp capping on maxillary first molars in mice, the effect of MTA on the histological characteristics of the dental pulp response was examined at different time points postoperatively. It was found that the inflammatory phase occurred in the first 2 days, then the healing process was initiated without inflammatory cell infiltration 2 weeks post operation, and the dentin bridge was visible with strong dentin sialophosphoprotein (DSPP) expression in odontoblast-like cells 5 weeks after the operation (Nirschl and Avery, 1983). Similarly, MTA displayed fewer inflammatory cell infiltrations and mild hard tissue deposition in the first week, while the dentin bridge with tubular structures was induced by MTA 4 weeks post operation when applied in pulp capping maxillary first molars from Wistar rats (Long et al., 2017). In addition, Park et al. (2014) discovered that the reparative dentin formation with minor pulp inflammation was observed 4 weeks after pulp capping in Wistar rats with MTA, which may be caused by the increased expression of odontogenic-related genes DSPP, dentin matrix protein 1 (DMP1), and osteocalcin (ON). On the other hand, MTA could inhibit the expression of IL-1α and IL-1β significantly and the dentin bridge was also formed at 30 days after capping in Sprague-Dawley rats (Kramer et al., 2014). Compared to rodent animal models, big animal models displayed more advantages in many aspects when studying disease (Wang et al., 2007). Therefore, Tabarsi et al. (2010) observed the dental pulp response when TA and calcium hydroxide (CH) were used in dental pulp capping for the premolar teeth of beagle dogs. The results showed that, compared to CH, MTA displayed a significantly better performance in pulp viability, pulp inflammation, and the formation of a calcified bridge. The pulp tissue underneath MTA contained odontoblast-like cells which was similar to health dental pulp tissue, whereas the odontoblast-like cells were absent when CH was applied. However, there are always tunnel defects that occur in dentin bridge formation in pulp capping with MTA (Tabarsi et al., 2010). It has been proven that the effect of MTA on dentin bridge formation may be caused by the odontoblastic differentiation of dental pulp stem cells (DPSCs) induced by Krüppel-like factor 5 (Han et al., 2017). In the meanwhile, given that glucose is a main source of energy for wound healing, Tohma et al. (2020) reported that glucose transporter 2 and 4 were involved in the odontoblastic differentiation and the related reparative dentin formation when MTA was used in rat pulp capping.

In order to enhance the effect of MTA on pulp capping *in vivo*, some studies focused on the discovery of novel substrates which could be used in combination with MTA in pulp capping. For example, the combined use of MTA with human placental extract in the pulp capping of rat maxillary first molars led to the superior formation of a dentin bridge and less of an inflammatory cell response (Chang et al., 2016). Besides, MTA with human placental extract significantly produced more of a calcific barrier than MTA alone, which was possibly caused by enhanced cell growth, odontoblastic differentiation, and angiogenesis in human DPSCs through the mTOR, MAPK, and NF-κB signaling pathway. When MTA was used in indirect pulp capping of canine teeth in beagle dogs, MTA induced the formation of reparative dentin with irregular features, whose quality was worse than reactionary dentin (Tziafas et al., 2000). To overcome this drawback, the copine7 (CPNE7) protein was used on the exposed dental surface and then MTA was applied on CPNE7 for indirect pulp capping (Choung et al., 2016). The combined use of the CPNE7 protein and MTA was able to induce typical reactionary dentin but not reparative dentin. The CPNE7 protein was secreted from dental epithelial cells and could promote the odontoblast differentiation, which implied that CPNE2 could improve the effect of indirect pulp capping with MTA.

As a new bioceramic in endodontics, Bioaggregate has a similar chemical composition with MTA but one of the main differences between them is that MTA contains bismuth oxide while Bioaggregate contains tantalum oxide as the radiopacifier (Park et al., 2010). Kim et al. (2016) examined the reparative dentin formation when MTA, Bioaggregate, and Biodentine were used in pulp capping maxillary first molars in Sprague-Dawley rats. The micro-CT analysis revealed that the dentin bridge induced by MTA or Biodentine was thicker than that by Bioaggregate, and the density of reparative dentin in Bioaggregate was lower than that in MTA or Biodentine. Furthermore, DSP expression was obviously higher in the MTA group compared to that in the Biodentine or Bioaggregate groups. The results suggested that MTA and Biodentine had superior dentinogenic potential compared to Bioaggregate that may be caused by the difference between bismuth oxide and tantalum oxide. Interestingly, it was reported that, similar to MTA, Biodentine could promote the formation of reparative dentin via odontoblastic differentiation of bone marrow-derived cells when used in pulp capping in mice, which implied that bone marrow-derived cells were also involved in reparative dentinogenesis (Frozoni et al., 2020). Paula et al. (2020) investigated the influence of MTA and Biodentine on pulp capping in the mandibular first molars of Wistar Han rats. It was shown that MTA induced moderate inflammatory infiltration 3 days post operation and the formation of mineralized tissue 21 days after the operation, whilst Biodentine induced a slight inflammatory infiltration 3 days post operation and the formation of mineralized tissue 7 days post operation. Therefore, compared to MTA, Biodentine could aggressively induce the formation of mineralized tissue in pulp capping (Paula et al., 2020). The result was consistent with another study which found that Wnt/β-catenin could be activated by Biodentine for dentin bridge formation but not by MTA or CH (Yaemkleebbua et al., 2019). Besides, Zaen et al. (2020) compared the effect of Biodentine, MTA, and CH on pulp capping in mongrel dogs, then the histological features were examined at 7 days and 3 months after the operation. The results showed that Biodentine displayed less of an inflammatory response than MTA and CH at 7 days, but CH led to an inflammatory response and tissue necrosis higher than the others at 3 months. In terms of the dentin bridge formation, no significant difference among them was observed at 7 days, but there was a significant difference at 3 months. The dentin bridge formation was thickest using Biodentine, then MTC, and CH induced the thinnest bridge formation. Due to the genetic and anatomic similarities between minipigs and human, Pedano et al. (2020) examined the influence of Biodentine on pulp capping in Göttingen minipigs and found that Biodentine induced a slight inflammatory response after 7 days while the dentin bridge formation occurred after 70 days.

In terms of iRoot BP Plus, Liu et al. (2015) investigated the effect of MTA and iRoot BP Plus on rat pulp capping. The results revealed that iRoot BP Plus and MTA induced similarly mild inflammation after 1 week and the dentin bridge formed after 4 weeks, but it seemed that iRoot BP Plus possessed a superior induction capacity compared to MTA (Liu et al., 2015). Okamoto et al. (2018) found that, when used in pulp capping in Wistar rats, the volume of reparative dentin formation in the MTA group was higher than that in the iRoot BP Plus group, but iRoot BP Plus induced a significantly higher dentin density and volume than MTA after 4 weeks. Besides, both of them induced the formation of a calcified bridge completely with no inflammation underneath the bioceramic layer 3 months after they were, respectively, used in pulp capping incisors of beagle dogs (Shi et al., 2016). Moreover, in terms of the tooth discoloration, MTA produced gray discoloration while iRoot BP Plus did not. Zhu et al. (2014a) reported that, compared to MTA, iRoot BP Plus induced a stronger expression of odontoblastic genes and focal adhesion molecules in pulp tissue beneath the dentin bridge when used in Wistar rat pulp capping, which was due to the superior potential of iRoot BP Plus to enhance the adhesion and migration of human DPSCs. The underlying mechanism could be explained by the fact that both of these two calcium silicate-based bioceramics had the equal ability to promote dental pulp repair via the activation of the ERK 1/2, JNK, and Akt signaling pathway, which could reinforce the formation of both focal adhesion and stress fiber assembly, as well as the migration of dental pulp cells (Zhang et al., 2015).

## Root Perforation Repair

Accident root perforation during root canal treatment results in communication between the pulp chamber and the periodontium, which causes a chronic inflammatory response and the loss of the alveolar bone. An ideal bioceramic should have the ability to seal the perforation site and promote associated tissue regeneration. MTA was first introduced into the repair of lateral perforation by Lee et al. (1993). MTA was considered as the golden standard for the repair of root perforation and various animal studies were carried out to assess the application of MTA in perforation repair in terms of its advantages, disadvantages, and modification. Silva et al. (2009) created an animal model of furcation perforation in rats to assess the tissue reaction *in vivo*. The results found that plenty of polymorphonuclear cells were observed at 14 days after surgery while an increased number of mononuclear cells were present at 21 and 28 days. Meanwhile, abundant collagen deposition and granulation tissue were found at 28 days. Based on the histological features of the furcation perforation mentioned above and in order to evaluate the immune response in furcation perforation site, Lara et al. (2015) measured the cytokine expression at 7, 14, and 21 days when MTA was used to repair furcation perforation in mice, and it was shown that MTA increased the expression of TNF-α, IFN-γ, and RANKL during the early period (at 14 days), while it increased the expression of IL-10 in the later period which might be for tissue repair. Besides the rodent animal model, MTA could promote calcified bridge formation and inhibit the epithelial infiltration in the furcation area in dogs (Samiee et al., 2010). MTA induced mild inflammation at 1 month which then decreased at 3 months, and finally there was no inflammation present at 6 months. In the meanwhile, the new cementum formation started at 1 month and was complete at 6 months (Yildirim et al., 2005). Likewise, when MTA was applied to repair the lateral root perforation in mongrel dogs, MTA could induce the cementum deposition which was represented by a basophilic layer between MTA and the periodontal connective tissue after 1 month, and induced an irregular cementum bridge with tunnel defects after 6 months. During the whole period, the periodontal ligament was free of inflammatory response (Holland et al., 2001).

To assess the effect of Biodentine on perforation repair *in vivo*, de Sousa et al. (2019) investigated the influence of MTA, Biodentine, and gutta-percha on the repair of furcation perforation in Wistar rats, it was found that both MTA and Biodentine were able to significantly decrease the inflammatory response compared to gutta-percha after 14 and 21 days. Moreover, the bone resorption was significantly inhibited by MTA or Biodentine after 21 days. In addition, only 30% of MTA and Biodentine samples revealed cementum repair, which might be due to that fact that the period of 21 days was too short to form the cementum bridge. During the whole period, there was no epithelial proliferation observed in the presence of MTA or Biodentine, which was consistence with the previous study which reported that MTA immediately used in perforation repair could inhibit the epithelium migration (Samiee et al., 2010). Therefore, it was suggested that MTA should be used in perforation repair immediately after the root perforation was created. When MTA was applied in lateral root perforation repair immediately or after 7 days in dogs, the delayed application of MTA in perforation led to the contamination of the perforation site and displayed worse repair compared to the immediate application of MTA (Ford et al., 1995; Tawfik et al., 2016). Even though calcium hydroxide paste was used as a bactericidal agent to remove the contamination of the perforation site, it did not improve the repair effect (Holland et al., 2007). Tawfik et al. (2016) reported that, compared to MTA, platelet rich fibrin (PRF) or platelet rich plasma (PRP) could significantly reduce the inflammation response and the vertical bone loss in delayed and contaminated perforation in dogs, which suggested that PRP and PRF might be an alternative to the repair of contaminated perforation. In terms of the canine animal model, Silva et al. (2017) investigated the impact of MTA, Biodentine, and gutta-percha on the repair of the furcation perforation which was made on the center of the pulp chamber floor in beagle dogs. It was shown that Biodentine and MTA could significantly induce the formation of biomineralization, accompanied by no bone resorption and fewer inflammatory cell infiltrations in the furcation region, while gutta-percha could not. Compared to Biodentine, MTA induced the complete sealing of furcation perforation more frequently, as well as exhibited greater thickness and a greater area of mineralized tissue formation. The transcriptional factor RUNX2 was considered as the possible factor for the osteoblast differentiation induced by MTA and Biodentine. Conversely, Cardoso et al. (2018) also compared their influence on furcation perforation repair in beagle dogs and then assessed the histological features after 4 months (). It was shown that both MTA and Biodentine had equivalent radiographic responses, as well as similar hard tissue resorption and repair. However, compared to MTA, Biodentine displayed less of an inflammatory response, less extruded material, and a stronger cement repair ability. The differing results among these studies might be related to the selection of experimental model, the perforation technique, and the evaluation criteria. For example, in the procedure of perforation repair, MTA overfilling should be avoided because it was reported that lateral perforation repaired with MTA had a better effect at the correct quantity (Holland et al., 2001). The possible explanation was that overfilling led to the extrusion of the cement into the perforation site which could prevent the integration of the bioceramics and new formed bone/cementum (Silva et al., 2010). However, all the studies considered that both MTA and Biodentine were appropriate bioceramics for furcation perforation repair.

## Root-End Filling

An excellent root-end filling material needs to possess good biocompatibility, excellent sealing ability, the desirable ability to inhibit pathogenic microorganisms, and a predominant capacity to promote periapical tissue healing (Yuan et al., 2010). Torabinejad et al. (1995) compared the influence of MTA and amalgam on the periradicular tissue reaction in dog’s teeth with induced apical periodontitis. The results displayed that, compared to amalgam, MTA showed less inflammatory infiltration and more fibrous capsules underneath MTA. In addition, new cementum formed on MTA but not on the surface of amalgam (Torabinejad et al., 1995). Likewise, Torabinejad et al. (1995) also investigated the impact of MTA and amalgam on the periradicular tissue reaction in monkey’s teeth without an apical lesion. It was found that MTA could induce the cementum formation on both root-end dentin and MTA while amalgam only induced it on root-end dentin. Furthermore, MTA produced no inflammatory response whilst amalgam induced a moderate inflammatory infiltration in the adjacent connective tissue (Torabinejad et al., 1997). The previous studies suggested that amalgam was no longer a suitable root-end filling material while MTA was biocompatible to periapical tissue and able to promote the cementum regeneration.

Walivaara et al. (2012) investigated the periapical tissue response to MTA, intermediate restorative material (IRM, Dentsply/Caulk, Milford, DE, United States), reinforced zinc oxide cement (Super EBA, Harry J Bosworth Co., Skokie, IL, United States), and gutta-percha in dog’s teeth without a periapical lesion. IRM is a zinc oxide-eugenol with polymer reinforcement while Super-EBA is a general purpose zinc oxide eugenol cement reinforced with ethoxy benzoic acid (EBA) which may be used in crown cementation, temporary dressing, or as a cavity liner. Compared to Super EBA and gutta-percha, both MTA and IRM induced better healing in the periapical tissue and less inflammatory infiltration. Moreover, the cement formation could be deposited on the root dentin surface in the presence of all four materials, whilst only MTA had the ability to induce new cementum formation on the material surface. Likewise, Bernabe et al. (2005) examined the influence of MTA, IRM, Super EBA, and Zinc oxide eugenol (ZOE, SS White Artigos Dentários Ltda., Rio de Janeiro, RJ, Brazil) in retrofilling pulpless dog’s teeth with induced apical lesions, and the results revealed that MTA, IRM, and Super EBA had the similar ability to affect histopathologic characteristics but displayed a significantly better performance compared to ZOE. Furthermore, only MTA had the ability to promote the new cementum formation underneath MTA after 6 months (Bernabe et al., 2005). In the meantime, Otani et al. (2011) examined the healing of apical periodontitis with retrograde-filling with MTA or EBA in beagle dogs. The results showed that both MTA and EBA had a similar potential to induce the bone regeneration. However, new cementum was deposited on both the dentin surface and MTA, whilst only on the dentin surface in the presence of EBA. In terms of the cementum formation on the dentin surface, MTA induced cementum formation more frequently in comparison with EBA (Otani et al., 2011). Baek et al. (2010) evaluated the periapical bone regeneration when a root-end cavity was retrograde-filled with MTA, amalgam, or Super EBA, respectively, after endodontic surgery in dog’s teeth with induced periapical lesions. The distance between newly regenerated bone and materials was measured and the results displayed that the mean distance in the MTA group was equal to the thickness of the periodontal ligament significantly less than the others (Baek et al., 2010). In terms of the inflammatory response, MTA and Super EBA induced less inflammatory infiltration while amalgam displayed an obvious inflammatory response, which might be related to the inferior apical sealing ability of amalgam compared to MTA or Super EBA (Davis et al., 2003). Furthermore, the cementum formation on the surface of a resected root end was significantly more than Super EBA or amalgam (Baek et al., 2005). These studies provided the evidence that, compared to conventional root-end filling materials, MTA could induce new cementum formation. In addition, MTA displayed the surprising capacity to enhance the healing of periapical tissue even in pathophysiological environments, such as an infected root canal and a periapical lesion.

Sealer 26 (ESPE, Seefeld, Germany) and Sealapex (Kerr Corporation, Romulus, Mich) are epoxy resin-based materials which contain calcium hydroxide, bismuth oxide, and epoxy resin. Tanomaru-Filho et al. (2006) performed apical surgery to create a root-end cavity in a dog’s tooth with induced apical periodontitis and filled the cavity with MTA, Sealer 26, and Sealapex. Then the animal was killed after 6 months and it was found that all these three materials displayed the similar potential to repair the apical and periapical tissues (Tanomaru-Filho et al., 2006). Diaket (ESPE, Seefeld, Germany) is a polyvinyl resin and was first used in root-end filling in 1986. Regan et al. (2002) compared the effect of MTA and Diaket on the regeneration of periradicular tissue in dog teeth without an apical lesion and assessed the histological features after 60 days. The results showed that both MTA and Diaket could similarly enhance the formation of new bone, cementum, and the periodontal ligament (Regan et al., 2002). It seemed that MTA and resin had comparable potential to promote the regeneration of periapical tissue. However, Tawil et al. (2009) investigated the influence of MTA, IRM, and Geristore on the response of a periapical lesion in a dog’s tooth after endodontic microsurgery and retrograde-filling. It was reported that, compared to the hybrid ionomer composite resin Geristore, both MTA and IRM revealed a superior potential in the healing of periapical tissue, which suggested that calcium silicate-based bioceramics were more suitable than resin for root-end filling. In addition, MTA displayed better healing than Geristore by histopathologic assessment, but no significant radiographic difference between them could be observed. This contradiction might be due to the limitation of radiographic evaluation which could be overcome by the application of other more advanced radiographic equipment such as cone-beam computed tomography (Chen et al., 2015).

Although MTA possessed lots of advantages in the application of root-end filling, MTA had some other drawbacks including unsatisfied setting time, difficulty to handle, and tooth discoloration. Therefore, other root-end filling materials were introduced to overcome the limitations of MTA. For example, Quick-Set (Avalon Biomed Inc., Bradenton, FL, United States), a calcium aluminosilicate cement, had a setting time as short as 12 min and was difficult to washout after placement. Kohout et al. (2015) compared the effect of Quick-Set and MTA on the reaction of periapical tissue in a dog’s tooth and performed the histologic analysis after 90 days. The results displayed that both MTA and Quick-Set could promote the regeneration of new bone, cementum, and the periodontal ligament, whilst MTA induced less of an inflammatory reaction compared to Quick-Set (Kohout et al., 2015). Compared to Quick-Set, Quick-Set2 (Avalon Biomed Inc., Bradenton, FL, United States), a newly formulated calcium aluminosilicate material, contains similar components but fewer free alumina than Quick-Set. Walsh et al. (2018) examined the impact of MTA and Quick-Set2 on the periapical tissue response in dog teeth, which found that they had the similar ability to promote the healing of periapical tissue but MTA induced higher dentin bridge quality than Quick-Set2. In terms of Biodentine, Tang et al. (2019) created a root-ending cavity in teeth without apical periodontitis through periradicular surgery in beagle dogs and filled the cavity with MTA or Biodentine. The results showed that Biodentine displayed a stronger sealing capacity than MTA and both of them could promote the bone regeneration of after 6 months (Tang et al., 2019).

## Regenerative Endodontic Procedures (REPs)

The management of immature necrotic teeth is a challenge to endodontists. Firstly, the root of an immature tooth is weak, short, and the root canal wall is thin, which make the immature tooth root easier to fracture. Secondly, traditional endodontic treatment could not clean the root canal space completely and provide excellent apical sealing. The apexification and apical barrier techniques are two general methods to treat immature necrotic teeth, but they could not promote root development (Nosrat et al., 2012). REPs were introduced to handle immature necrotic teeth due to the capacity of the apical papilla stem cells which can promote the pulp regeneration and root development. Generally, the procedures begin with root canal irrigation with 2.5–5.25% NaOCl and 3% hydrogen peroxide, followed by intracanal medication with minocycline, ciprofloxacin, and metronidazole. After 1–4 weeks, the intracanal medication is removed and the periapical tissue is irritated to fill the root canal space with blood. Then MTA is placed over the blood clot to seal the root canal space (Huang, 2008). MTA is used for REPs due to its admirable microleakage-proof property and biocompatibility. Besides, MTA could promote the osteoblastic differentiation of apical papilla stem cells (Miller et al., 2018). REPs have been successfully carried out in different animals, such as dogs (Wang et al., 2010; Zhang et al., 2014; Rodriguez-Benitez et al., 2015; Saoud et al., 2015; Moradi et al., 2016; Stambolsky et al., 2016; Ghoddusi et al., 2017; Palma et al., 2017), sheep (Altaii et al., 2017), and ferrets (Torabinejad et al., 2014, 2015, 2018). To clarify what type of new-grown tissue is present REPs *in vivo*, Wang et al. (2010) found that bone/cementum-like tissue and regenerated soft tissue could be observed in the root canal during REPs in dogs. Both bone-like and periodontal ligament-like tissue were localized in the central area of the root canal, while cementum-like tissue was deposited on the inner canal surface which led to the thickening of the dentinal wall and the increase of the root length (Wang et al., 2010). Furthermore, it was reported that cementum-like tissue in the apical portion was more developed than in the coronal portion covered by MTA, which suggested that REPs might start from the apical area of the root and then continue along the root coronally (Altaii et al., 2017). As to the regenerated soft tissue in REPs, it could only be described as pulp-like tissue which is not the real pulp tissue because of the absence of an odontoblast layer (Zhang et al., 2014). To solve this problem, Torabinejad et al. (2018) found that the pulp dentin complex could be regenerated if the apical pulp was not damaged, while complete pulp removal resulted in the production of bone-like tissues. In terms of MTA, a mineralized bridge was always observed underneath the MTA and the bridge appeared to consist of cementum-like tissue, which might be due to the osteoinductive activity of the MTA (Wang et al., 2010). Similarly, Saoud et al. (2015) reported that two kinds of mineralized bridges were observed underneath the MTA in REPs in dogs according to the components of the bridges. One type was similar to bone but not to dentin due to the absence of the tubular structure in dentin. The fibroblasts, mesenchymal cells, and collagen fibers were attached underneath the mineralized bridge. The other type was mainly composed of cementoblasts and cementocytes which could be observed above the mineralized bridge (Saoud et al., 2015).

## Apexification

If REPs failed in the treatment of immature necrotic teeth, apexification could be a suitable option to achieve an excellent apical sealing. Calcium hydroxide has been used for apexification over the past few decades. However, calcium hydroxide possesses several drawbacks such as unsatisfactory apical closure and delayed treatment. Due to its predominant sealing effect and good biocompatibility, MTA was introduced into apexification as a potential apical seal material. Shabahang et al. (1999) compared the influence of MTA and calcium hydroxide on apical closure in the immature necrotic teeth of dogs. The results displayed that, compared to calcium hydroxide, MTA induced more apical closure, more hard tissue formation, and less inflammatory infiltration (Shabahang et al., 1999), which was in line with another studies that investigated the effect of MTA and calcium hydroxide on apexification in monkey teeth (Ham et al., 2005). To investigate the newly regenerated tissue in apexification, Palma et al. (2017) evaluated the newly generated tissue histologically when MTA was used for apexification in immature teeth with periapical lesion in dogs. The results revealed that MTA could induce not only the resolution of periapical lesions but also the apical closure via the formation of a cellular cementum bridge surrounded by the periodontal ligament. Furthermore, another kind of newly mineralized tissue, mainly composed of dentin and cementum, was localized underneath the cellular cementum bridge closely and surrounded by an extension of the periodontal ligament, which histologically contributed to the augmentation of root length. In addition, MTA overfilling could cause the failure in the formation of the cellular cementum bridge, which might be due to the previous use of calcium hydroxide paste ahead of apical sealing with MTA (Felippe et al., 2006).

## Conclusion and Perspective

So far there have been plenty of studies to investigate the biocompatibility and bioactivity of calcium silicate-based bioceramics in endodontics. MTA is by far the calcium silicate-based bioceramic which has been investigated most thoroughly and is considered the gold standard in endodontic applications due to its excellent physicochemical properties and biological characteristics. However, in comparison with MTA, there are not enough *in vivo* studies to assess the biocompatibility and bioactivity of other calcium silicate-based bioceramics such as Bioaggregate, Biodentine, and especially iRoot BP/FS/SP in endodontics. Therefore, more *in vivo* studies are required in future. Furthermore, the observations and results from various animal models, such as mice, rat, sheep, dog, and ferret are inconsistent due to several reasons. Firstly, the (patho-)physiological and anatomical differences among different animals may lead to a discrepancy in the results from different studies. Secondly, due to the fact that various experimental procedures and the related parameter assessment criteria are distinctive, the results and the subsequent conclusions may be influenced by these differences. Therefore, it is hard to compare the results from different studies so that they cannot be directly applied to human beings. It is essential to establish a well-defined gold standard of animal models and the related experiment procedures as well as the parameter evaluation to overcome this flaw. On the other hand, although calcium silicate-based bioceramics displayed excellent biocompatibility and bioactivity, the combined use of calcium silicate-based bioceramics with other materials/procedures can improve the efficiency of calcium silicate-based bioceramics in endodontics. For example, the addition of the CPNE7 protein into MTA could induce the formation of reactionary dentin while MTA only could induce the formation of reparative dentin with irregular features when used in pulp capping (Choung et al., 2016). In addition, the combined application of photobiomodulation therapy with MTA could significantly improve apexification in necrotic rat molars with open apex compared to that of MTA alone (Zaccara et al., 2019). Finally, it has been reported that systemic disease can influence the efficiency of MTA in endodontics. For instance, diabetes mellitus could inhibit mineral differentiation in subcutaneous implantation (de Azevedo et al., 2018) and dentin bridge formation in rat pulp capping (Garber et al., 2009). So when calcium silicate-based bioceramics are applied in patients with systemic disease, the control and treatment of the systemic disease should be carried out ahead of any procedures to improve the success rate of calcium silicate-based bioceramics in endodontics.

## Author Contributions

WS conceived and wrote the manuscript. WS and WS contributed to data acquisition, analysis, and interpretation. LC and ZY critically revised the manuscript.

## Conflict of Interest

The authors declare that the research was conducted in the absence of any commercial or financial relationships that could be construed as a potential conflict of interest.
